# The fibro‐ and cyto‐architecture demarcating the border between the dentate gyrus and CA3 in sheep (*Ovis aries*) and domestic pig (*Sus scrofa domesticus*)

**DOI:** 10.1002/hipo.23457

**Published:** 2022-08-01

**Authors:** Jan Sigurd Blackstad, Kirsten K. Osen, Trygve B. Leergaard

**Affiliations:** ^1^ Department of Molecular Medicine, Institute of Basic Medical Sciences University of Oslo Oslo Norway; ^2^ Kavli Institute for Systems Neuroscience and Center for Biology of Memory Norwegian University of Science and Technology Trondheim Norway

**Keywords:** border description, CA3, comparative anatomy, dentate gyrus, Golgi impregnation, hippocampus, mossy fiber system, Timm‐staining

## Abstract

The hippocampal formation is essential for spatial navigation and episodic memory. The anatomical structure is largely similar across mammalian species, apart from the deep polymorphic layer of the dentate gyrus and the adjacent part of cornu ammonis 3 (CA3) which feature substantial variations. In rodents, the polymorphic layer has a triangular cross‐section abutting on the end of the CA3 pyramidal layer, while in primates it is long and band‐shaped capping the expanded CA3 end, which here lacks a distinct pyramidal layer. This structural variation has resulted in a confusing nomenclature and unclear anatomical criteria for the definition of the dentate‐ammonic border. Seeking to clarify the border, we present here a light microscopic investigation based on Golgi‐impregnated and Timm–thionin‐stained sections of the Artiodactyla sheep and domestic pig, in which the dentate gyrus and CA3 end have some topographical features in common with primates. In short, the band‐shaped polymorphic layer coincides with the Timm‐positive mossy fiber collateral plexus and the Timm‐negative subgranular zone. While the soma and excrescence‐covered proximal dendrites of the mossy cells are localized within the plexus, the peripheral mossy cell dendrites extend outside the plexus, both into the granular and molecular layers, and the CA3. The main mossy fibers leave the collateral plexus in a scattered formation to converge gradually through the CA3 end in between the dispersed pyramidal cells, which are of three subtypes, as in monkey, with the classical apical subtype dominating near the hidden blade, the nonapical subtype near the exposed blade, and the dentate subtype being the only pyramidal cells that extend dendrites into the dentate gyrus. In agreement with our previous study in mink, the findings show that the border between the dentate gyrus and the CA3 end can be more accurately localized by the mossy fiber system than by cyto‐architecture alone.

## 1 INTRODUCTION

The dentate gyrus (DG) and the cornu ammonis 3 (CA3) are two adjacent allocortical areas in the hippocampal formation of mammalian brains with particular importance for spatial navigation and episodic memory (Leutgeb et al., 2007; Maguire et al., 1997; Moser et al., 2017; O'Keefe & Nadel, 1978; Scoville & Milner, 1957; Senzai, 2019; Squire, 1992). The two areas are integral parts of the parahippocampal‐hippocampal circuitry that forms a largely unidirectional, multisynaptic neuronal pathway from the entorhinal cortex in the parahippocampal region through the hippocampal formation and back again (Amaral & Witter, 1989; Cappaert et al., 2015; van Strien et al., 2009; Witter et al., 1989).

The DG connects to CA3 by mossy fiber axons from DG granule cells (Blackstad et al., 1970; Blackstad & Kjaerheim, 1961; Claiborne et al., 1986; Gaarskjaer, 1978; Lorente de Nó, 1934; Ramón y Cajal, 1911; Swanson et al., 1978). The granule cell dendrites, in the molecular layer, are supplied by perforant path fibers from the entorhinal cortex of the parahippocampal region (Blackstad, 1958; Hjorth‐Simonsen, 1972; Hjorth‐Simonsen & Jeune, 1972; Steward, 1976), while the mossy fibers, after having emitted collaterals (Golgi, 1886) to the deep polymorphic layer of the DG (Acsády et al., 1998; Claiborne et al., 1986), proceed to the CA3 were they are oriented in parallel, perpendicular to the longitudinal axis of the hippocampal formation (Andersen et al., 1969; Andersen et al., 1971; Blackstad et al., 1970), being unbranched with *én passant* collaterals like axons of other granule cell systems (D'Angelo et al., 2011; Mugnaini, Warr, & Osen, 1980; Osen et al., 1995; Ramón y Cajal, 1909; Ramón y Cajal, 1911; Voogd & Glickstein, 1998). Mossy cells of the polymorphic layer, by their projection to the inner third of the molecular layer (Blackstad, 1956; Blackstad, 1985; Laurberg, 1979; Laurberg & Sorensen, 1981; Soriano & Frotscher, 1994; Swanson et al., 1981; Zimmer, 1971), constitute a modulatory feedback link to the granule cells (Buckmaster et al., 1996; Ribak et al., 1985; Scharfman & Myers, 2012), while pyramidal cells of the CA3 have a widespread collateral projection to the CA1 (Ishizuka et al., 1990; Schaffer, 1892), which in turn projects to the entorhinal cortex, partly via the subiculum, thus completing the circuitry (Amaral & Witter, 1989; Cappaert et al., 2015; van Strien et al., 2009; Witter et al., 1989).

Even though the hippocampus as a whole appears surprisingly similar across mammalian species, comparative studies are complicated by significant species differences in the shape and relative size of the DG and the CA3 (Amaral et al., 2007; Seress, 2007). A common understanding of how the DG and the CA3 can be reproducibly identified in different species is, therefore, of crucial importance for our ability to compare and integrate results across experimental studies.

The location of the DG/CA3 border has long been a matter of dispute, not least because of the transformation of the DG polymorphic layer from a triangular cross‐section (usually referred to as the hilus), abutting on the end of the CA3 pyramidal layer in the much‐studied rat (Amaral et al., 2007; Haug, 1974), to a long, thin band capping the expanded nonstratified CA3 end in primates (Buckmaster & Amaral, 2001; Lavenex & Amaral, 2007; Kondo et al., 2008). The descriptive term “CA3 end”, which is used here for the DG‐near end of the CA3, corresponds approximately to the terms “proximal CA3” (Ishizuka et al., 1990) and “CA3c” (Lorente de Nó, 1934). The long, curved, and twisted shape of the hippocampus as a whole adds to the difficulties in distinguishing the DG/CA3 border in conventional planes of sectioning.

Historical examples of arbitrary DG/CA3 border definitions, not based on cyto‐ or fibro‐architecture, include (1) the classical Golgi‐study of Lorente de Nó (1934) in mouse, rabbit, monkey, and man, in which the polymorphic layer is regarded as an extension of the Ammon's horn termed “CA4”, and (2) a series of histochemical studies in domestic pig (Holm & Geneser, 1989; Holm & Geneser, 1991a, 1991b), guinea pig (Jensen, 1975), and rabbit (Geneser, 1987a, 1987b), in which the CA3 end is included in an extended DG named “area dentata”. In these examples, the different connectivity's of the CA3 and the polymorphic layer were apparently not taken into account.

Thanks to detailed cytoarchitectural studies in rat (Amaral, 1978) and monkey (Buckmaster & Amaral, 2001), there is now a general agreement about the key cytological features of the DG and the CA3 (Amaral et al., 2007; Seress, 2007). Yet, a precise and operational definition of the DG/CA3 borderline that is applicable across species, is still missing (Cappaert et al., 2015). Since part of the problem may be ascribed to certain cytoarchitectonic similarities between the polymorphic layer and the unstratified expanded CA3 end, we here propose to base the DG/CA3 borderline on the mossy fiber collateral plexus rather than on the cytoarchitecture alone.

To elucidate this option, we investigated the DG/CA3 border in the Artiodactyla sheep and domestic pig, in which the band‐shaped polymorphic layer and the subjacent expanded nonstratified CA3 end have many features in common with monkey (Buckmaster & Amaral, 2001; Kondo et al., 2008). To our knowledge, the only available morphological descriptions of the sheep hippocampus are the study by Rose (1942) and a study of fetal sheep by Godina and Barasa (1964). Although Rose (1942) reported on some basic features of the structure, we here may provide the first detailed anatomical description of the DG and CA3 end in adult sheep. A reappraisal of the DG/CA3 border in pig was also warranted, since previous studies in this species, as mentioned above (Holm & Geneser, 1989; Holm & Geneser, 1991a, 1991b), are at odds with the currently accepted distinction between the DG and the CA3 (Amaral et al., 2007; Seress, 2007).

We utilized a sparse, but unique archive histological material prepared by the late T.W. Blackstad and the late F. Geneser for comparison of Timm–thionin‐ and Golgi‐stained sections from adjacent blocks of the same hippocampus, cut perpendicular to its curved long axis, that is, approximately in parallel with the main mossy fibers and the so‐called hippocampal lamellae (Blackstad et al., 1970; Sloviter & Lømo, 2012). The Golgi‐method, besides staining cell bodies and dendrites of randomly sampled neurons, stains unmyelinated axons like the mossy fibers and their collaterals, while the Timm‐method (Danscher, 1981; Haug, 1973; Timm, 1958) relatively specifically stains the zinc containing terminals, which occur scattered along the entire length of both main mossy fibers and their collaterals, thus making them distinguishable and traceable to a certain extent. The combination of Timm‐ and thionin‐staining enabled a direct comparison between cyto‐ and fibro‐architecture in the same tissue section.

Our findings support the use of the mossy fiber collateral plexus in localizing the DG/CA3 borderline, which in principle also should provide a general key to comparing the two adjacent areas across mammals.

## 2 MATERIALS AND METHODS

The present study is based on a historical histological material created from sheep and domestic pig hippocampi by the late professor Theodor W. Blackstad (1925–2003), in collaboration with the late professor Finn Geneser (1938–2018), who permitted the use of the material (F. Geneser, personal communication to K.K. Osen, 2003). Their notes on several initial observations have served as an inspiration and starting point for the present study.

The materials include the hippocampi of two 8‐week‐old sheep (*Ovis aries*, TBL75 and TBL76) obtained by T. W. Blackstad from the Norwegian School of Veterinary Science (Dal, Asker, Norway), and three domestic pigs (Sus scrofa, TBL80, FIJ2, FIJ3) procured by F. Geneser at the University of Aarhus, Denmark. The brains were processed using standard laboratory routines employed at the Universities of Oslo and Aarhus in the 1980s, with local institutional approvals in line with national legislation and practice at that time. For information about animals and histological processing, as summarized below, we have relied on notes and protocols left by T.W. Blackstad. The protocols of F. Geneser are no longer available.

Both sheep were euthanized with an overdose of pentobarbital. Within 1 h, the brains were removed and immersed in 3% glutaraldehyde and 0.4% sodium sulphide in 0.15 M Sørensen phosphate buffer for 10 h. During this period each hemisphere was gradually dissected to expose the hippocampus to view bilaterally, leaving some minor physical artifacts at the pial surface in some sections. Each hippocampus was subdivided into five blocks cut perpendicular to the curved long septotemporal axis of the hippocampus, excluding the extreme ends. The blocks were labeled a–e, starting from septal. Blocks a, c, and e were used for Golgi‐staining, and blocks b and d for Timm–thionin‐staining (see below).

The brain of one domestic pig, perfusion‐fixed at the University of Aarhus, was shipped to Oslo in an aldehyde solution for further processing. The left hippocampus was exposed and cut perpendicular to its long axis in five blocks numbered 1–5 from septal to temporal. All blocks were subjected to Golgi‐staining together with the sheep material (see below).

Tissue blocks of both species destined for Golgi‐staining (those from sheep after 10 h in the sulphide solution) were immersed in 1% paraformaldehyde in 0.1 M Sørensen phosphate buffer, pH 7.4, for 10 days, followed by 2.4% potassium dichromate with 0.2% osmium tetroxide for 2 days, and then 3.0% potassium dichromate for a few days. After covering the blocks with 2–4% agar, silver impregnation was carried out with 0.75% silver nitrate for 2 days. Finally, the agar was removed before dehydration and embedding in Araldite. Sections 100 μm thick were cut on a sliding microtome with a hot steel knife.

Tissue blocks of sheep destined for Timm–thionin‐staining were, after 10 h in sulphide solution, immersed in 30% sucrose in 0.1 M sodium phosphate buffer at pH 7.4 for another 13–15 h, and cut into 40 μm thick sections using a cryostat. Sections were mounted and subjected to Timm‐staining (Holm & Geneser, 1989) before being counterstained with thionin to visualize cell bodies.

All sections were cut perpendicular to the long axis of the hippocampus, in the following referred to as the perpendicular plane, which is approximately parallel to the so‐called lamellar plane (Andersen et al., 1969; Andersen et al., 1971; Blackstad et al., 1970; Sloviter & Lømo, 2012). Because of the curvature of the hippocampus, the orientation of the sections with respect to the axes of the brain differs between the blocks. In addition, three thionin‐stained sections and three Timm–thionin‐stained sections from two other pigs were received from the University of Aarhus in the late 1980s. These ~40 μm thick sections had apparently been cut horizontally with the hippocampus in situ in the brain, not adjusting for the curvature and orientation of the hippocampus, and thus not exactly perpendicular to its long axis. Details of the Timm‐method used in these cases are not available.

**FIGURE 1:**
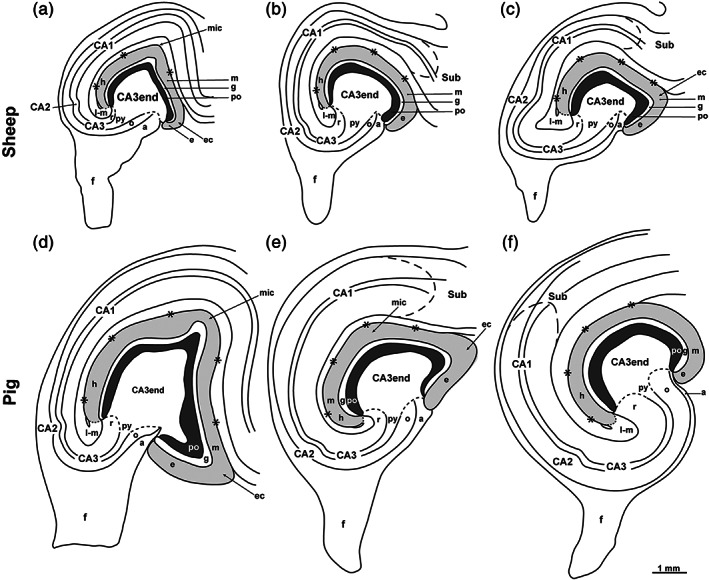
Diagrams showing the dentate gyrus/cornu ammonis in sheep and pig. Camera lucida drawings of the DG and the cornu ammonis in sheep (a–c) and pig (d–f) based on Golgi‐stained sections cut perpendicular to the long septotemporal axis of the hippocampus at septal (a, d), middle (b, e), and temporal (c, f) levels in both species, in sheep supplied by Timm–thionin‐stained sections from alternating tissue blocks. Being cut from different blocks, the section drawings are arbitrarily aligned with respect to each other. The hippocampal fissure is marked by asterisks, the border between the DG molecular layer (m, shaded gray) and the lacunar‐molecular layer (l‐m) is marked by a dotted line while the curved dashed line indicates the CA1/Subiculum (Sub) border. The end of the visibly laminated stratum radiatum, stratum oriens, and alveus is marked by stippled lines. a, alveus; CA1–CA3, cornu ammonis area 1–3; e, exposed blade; ec, exposed crest; f, fimbria; g, granular layer; h, hidden blade; mic, middle crest; o, oriens layer; po, polymorphic layer; py, pyramidal layer; r, radiate layer.

High‐resolution microscopic images were acquired at multiple depths of focus using an automated slide scanner system (Axio Scan Z1, Cark Zeiss MicroImaging, Jena, Germany) employing the extended depth of focus function module provided in the Zen software (Carl Zeiss MicroImaging, Jena, Germany). The ensuing 2‐D images show cellular structures from different depths in the section projected into one plane, thus hampering differentiation between various focus depths and making strongly stained adjacent elements appear to merge. Septotemporal differences are reflected in the diagrams of Figure 1, but were otherwise not evaluated.

## 3 RESULTS

We here present observations on the cyto‐ and fibro‐architecture of the DG and CA3 end in sheep and pig, with special emphasis on the mossy fiber system and its significance for the definition of the borderline between the polymorphic layer and the subjacent expanded CA3 end, here referred to as the DG/CA3 border. Considering the mossy fiber system as the principal organizing element, the polymorphic layer was defined as the area covered by the plexus of mossy fiber collaterals plus the superjacent subgranular zone free of zinc‐containing mossy fiber terminals, observed previously among others in guinea pig (Geneser‐Jensen et al., 1974) and rabbit (Geneser, 1987b) under the name “outer plexiform layer”. The term CA3 end, on the other hand, was used for the area of scattered pyramidal cells deep to the mossy fiber collateral plexus, found also in monkey and human (Amaral et al., 2007; Buckmaster & Amaral, 2001; Kondo et al., 2008; Lavenex et al., 2007). This cellular area proved to coincide with the area of scattered main mossy fibers gradually converging into the dense mossy fiber bundle adjacent to the pyramidal layer of the main CA3. The present definition of the DG/CA3 borderline is compatible with the generally accepted perception of the border (Amaral et al., 2007), while at the same time it underlines the relationship of the polymorphic layer with the mossy fiber collaterals and of the CA3 end with the main mossy fibers (Acsády et al., 1998; Claiborne et al., 1986). It also supports the suitability of the Timm‐staining method for recognition of the DG/CA3 borderline in cases where this is difficult to set by the cytoarchitecture alone. Basing the borderline definition on the mossy fiber system, however, required a clarification of the topographic relationships of the somata and dendrites of the DG mossy cells and the CA3 pyramidal cells to the DG/CA3 borderline, as also focused on in our previous study in mink (Blackstad et al., 2016).

Below, we first compare the overall organization of the DG and CA3 end in sheep and pig. We then describe the cyto‐and fibro‐architecture of the polymorphic layer and the CA3 end in the two species separately, with emphasis on the sheep, which has been subject to fewer hippocampal studies than the pig. Our discussion and conclusions build on observations from both species supplemented by findings reported in the literature.

### 3.1 Overall organization of the DG and CA3 end in sheep and pig

Figure 1 presents diagrams of the sheep and pig hippocampus, oriented perpendicular to its curved longitudinal axis, at three comparative septotemporal levels. The diagrams were constructed from *camera lucida* drawings of Golgi‐stained sections, aided by Timm–thionin‐sections to localize the deep border of the polymorphic layer and the transition of the CA3 end to the main CA3. The pattern appeared largely similar in the two species, both featuring a three‐layered DG with a slim polymorphic layer (down to about 200 μm thick) deep to the molecular and granular layers. The three layers of the DG together form a concavity filled by the expanded, nonstratified CA3 end, much like the situation in monkey (Buckmaster & Amaral, 2001). Unfortunately, the concavity with the expanded CA3 end is often referred to as the hilus (Holm & Geneser, 1991b) or the hilar region (Amaral et al., 2007), which is confusing since the term hilus is originally used in rodents for the polymorphic layer. In sheep and pig, the cell‐rich CA3 end gradually tapers into the pyramidal layer of the main CA3 flanked by the cell poor radiate and oriens layers. The transition appeared more gradual in sheep, in which the entire hippocampal region appeared smaller, in agreement with the generally ~40% smaller brain weight compared to pig (Minervini et al., 2016; Steinhausen et al., 2016).

In both sheep and pig, the molecular layer, which runs deep to the obliterated hippocampal fissure (Figure 1, asterisks), is contiguous with the CA3 lacunar‐molecular layer close to the U‐turn of the latter near the bottom of the fissure (Figure 1, dotted borderline between the two). Compared to mink (Blackstad et al., 2016, their fig. 1), the DG in sheep and pig features a more rounded surface with less marked crests apart from an exposed crest at the outlet of the hippocampal fissure and a middle crest at the septal level (Figure 1a,d) reminiscent of the V‐shaped DG at this level in rat (Amaral et al., 2007; Boccara et al., 2015; Kjonigsen et al., 2011; Kjonigsen et al., 2015). Despite the lack of a distinct hidden crest, we roughly divide the DG in an exposed, middle and hidden blade for descriptive purposes, and in the following use these terms to indicate approximate locations within the DG and the CA3 end.

### 3.2 Cyto‐ and fibro‐architecture of the polymorphic layer and CA3 end in sheep

**FIGURE 2:**
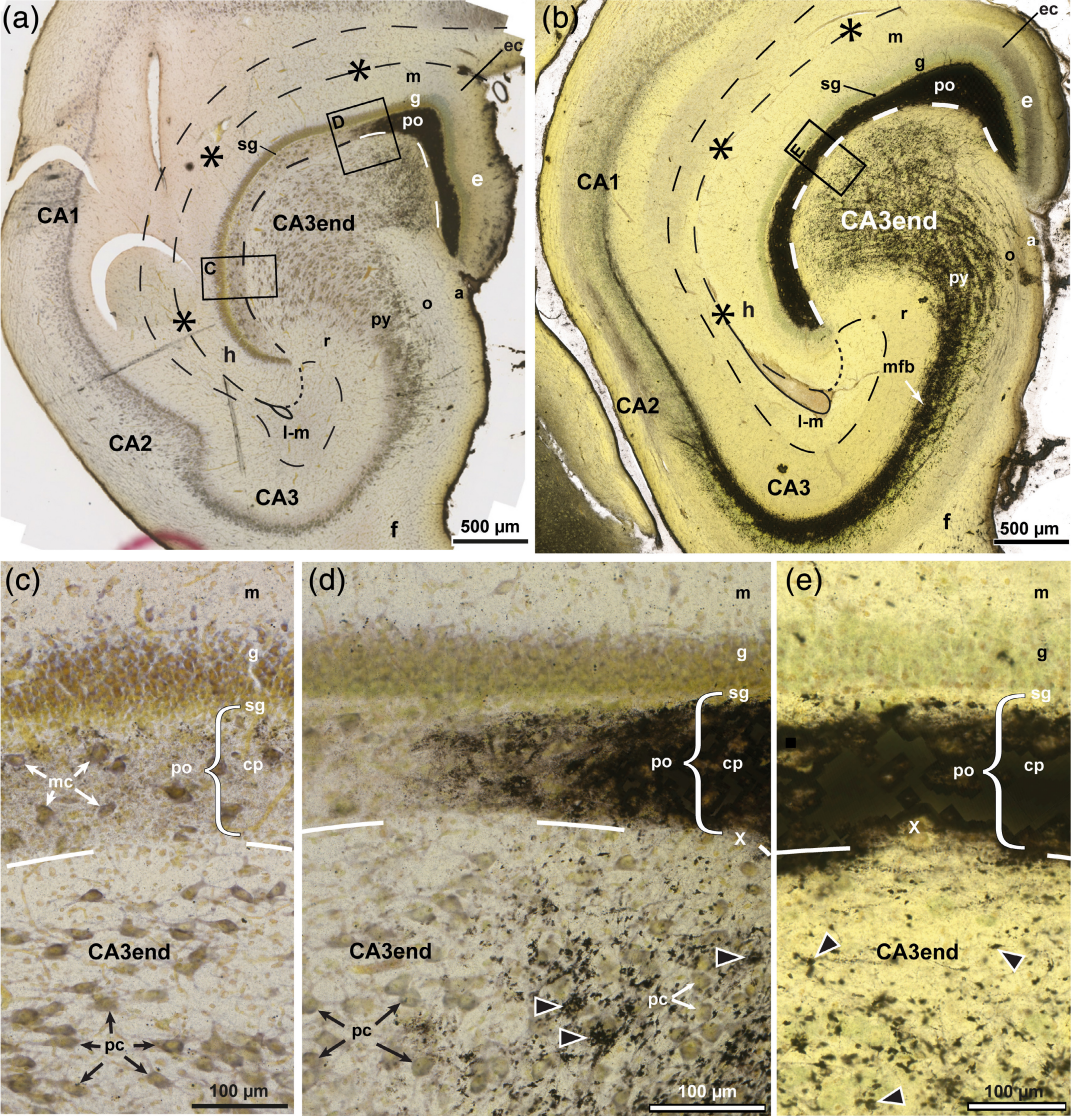
The sheep dentate gyrus and CA3 end in Timm–thionin‐staining. Photomicrographs of Timm–thionin‐stained sections cut perpendicular to the approximate middle of the long septotemporal axis of the hippocampus, (a, c, d) with predominance of thionin‐staining except in the most exposed part. (c–e) Higher magnifications of the boxed areas in (a, b). In (b), a trace of the three‐partition of the molecular layer (m) is seen in the exposed blade (e). The polymorphic layer (po) consists of the Timm‐positive mossy fiber collateral plexus (cp) and the Timm‐negative subgranular zone (sg) (a–e). The hippocampal fissure is marked by asterisks and dashes, and a loop in the deep end, the po/CA3 border, and the lacunar‐molecular layer (m‐l) by dashed lines, apart from the dotted border with the molecular layer (m). while mossy fiber giant terminals are marked by black arrowheads. a, alveus; CA1–CA3, cornu ammonis area 1–3; e, exposed blade; ec, exposed crest; f, fimbria; g, granular layer; h, hidden blade; mc, mossy cells; mfb, mossy fiber bundle; o, oriens layer; pc, pyramidal cells; py, pyramidal layer; r, radiate layer; x, excavation in the cp.

**FIGURE 3:**
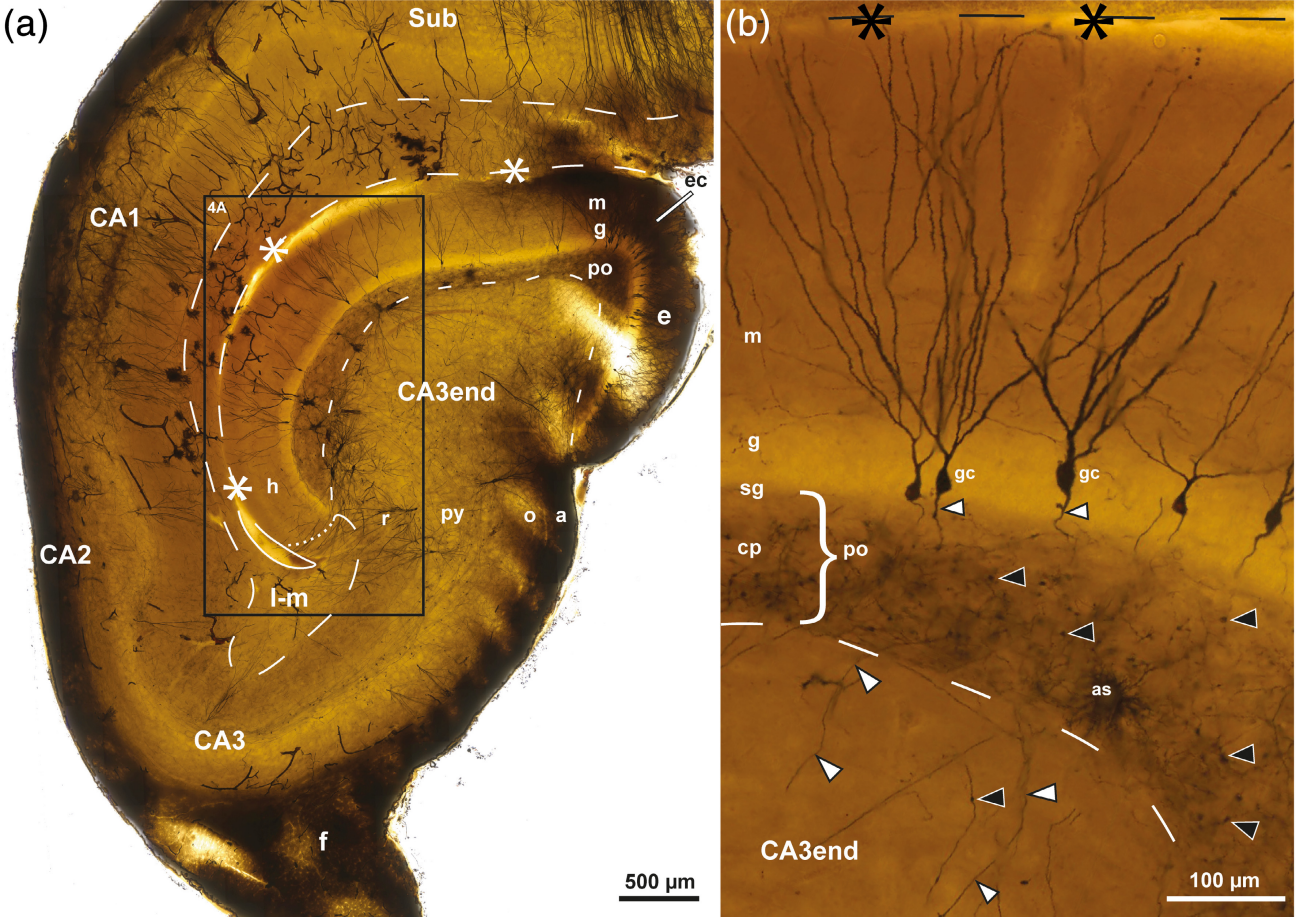
Mossy fiber collateral plexus in Golgi‐sections of sheep. Photomicrographs of Golgi‐stained sections cut perpendicular to the long septotemporal axis of the hippocampus, at temporal (a) and middle (b) levels. The boxed area in (a) is shown at higher magnification in Figure 4a. (b) Features the three layers of the DG: the molecular layer (m), the granular layer (g), the polymorphic layer (po) with the subgranular zone (sg) and the mossy fiber collateral plexus (cp), and some of the subjacent CA3 end. The granule cells (gc) differ with respect to dendritic thickness and soma size. Mossy fibers are marked by white arrowheads, mossy fiber giant terminals by black arrowheads, the hippocampal fissure by asterisks and dashes, and a loop in the end, the po/CA3 border, and the lacunar‐molecular layer (m‐l) by dashes, apart from the dotted border with the m. a, alveus; as, astrocyte; CA1–CA3, cornu ammonis area 1–3; e, exposed blade; ec, exposed crest; f, fimbria; h, hidden blade; o, oriens layer; py, pyramidal layer; r, radiate layer; sg, subgranular zone; Sub, subiculum.

The spatial organization of mossy fibers and related cells in sheep were observed in Timm–thionin‐ and Golgi‐stained sections, oriented in the perpendicular plane (Figures 2 and 3). In the lack of a plain thionin‐stained section in sheep, we utilized a very special Timm–thionin‐stained section, in which the cellular pattern was clearly visible because of a failure of the Timm‐staining throughout most of the section except in and near to the exposed blade of the DG (Figure 2a,c,d). The stronger Timm‐staining of the subpial brain tissue observed in some cases (Figure 2a,b) may be due to a more direct exposure to the sulphide solution peripherally in the block.

#### 3.2.1 Cytoarchitecture of the sheep DG and CA3 end in thionin‐stained sections

In the thionin‐dominated part of the above‐mentioned section, the densely packed granular layer appeared sharply delimited against the polymorphic layer, which, apart from the narrow subgranular zone, exhibited scattered somata of large multipolar neurons perceived by us as mossy cells (Figure 2a,c,d). The CA3 end was composed of two indistinctly separated zones, a narrow cell poor zone next to the polymorphic layer and a larger cell rich zone gradually tapering into the CA3 pyramidal layer. The CA3 cells, perceived by us mostly as pyramidal cells, appeared slightly smaller and in part more oval than the mossy cells. In the hidden part, the cells tended to be elongated and oriented with their long axis across the extension of the long axis of the pyramidal layer, which here curved toward the hidden blade (Figure 2a,c), in agreement with the illustrations of Godina and Barasa (Godina & Barasa, 1964, their fig. 9) in fetal sheep. In the exposed part of the CA3 end, the cells appeared more multipolar (Figure 2a,d).

The polymorphic layer and the CA3, besides the mossy cells and the pyramidal cells, contained numerous smaller interneurons (Amaral, 1978; Freund & Buzsáki, 1996), which were not further considered in our study.

#### 3.2.2 Architecture of the sheep mossy fiber system in Timm‐ and Golgi‐staining

**FIGURE 4:**
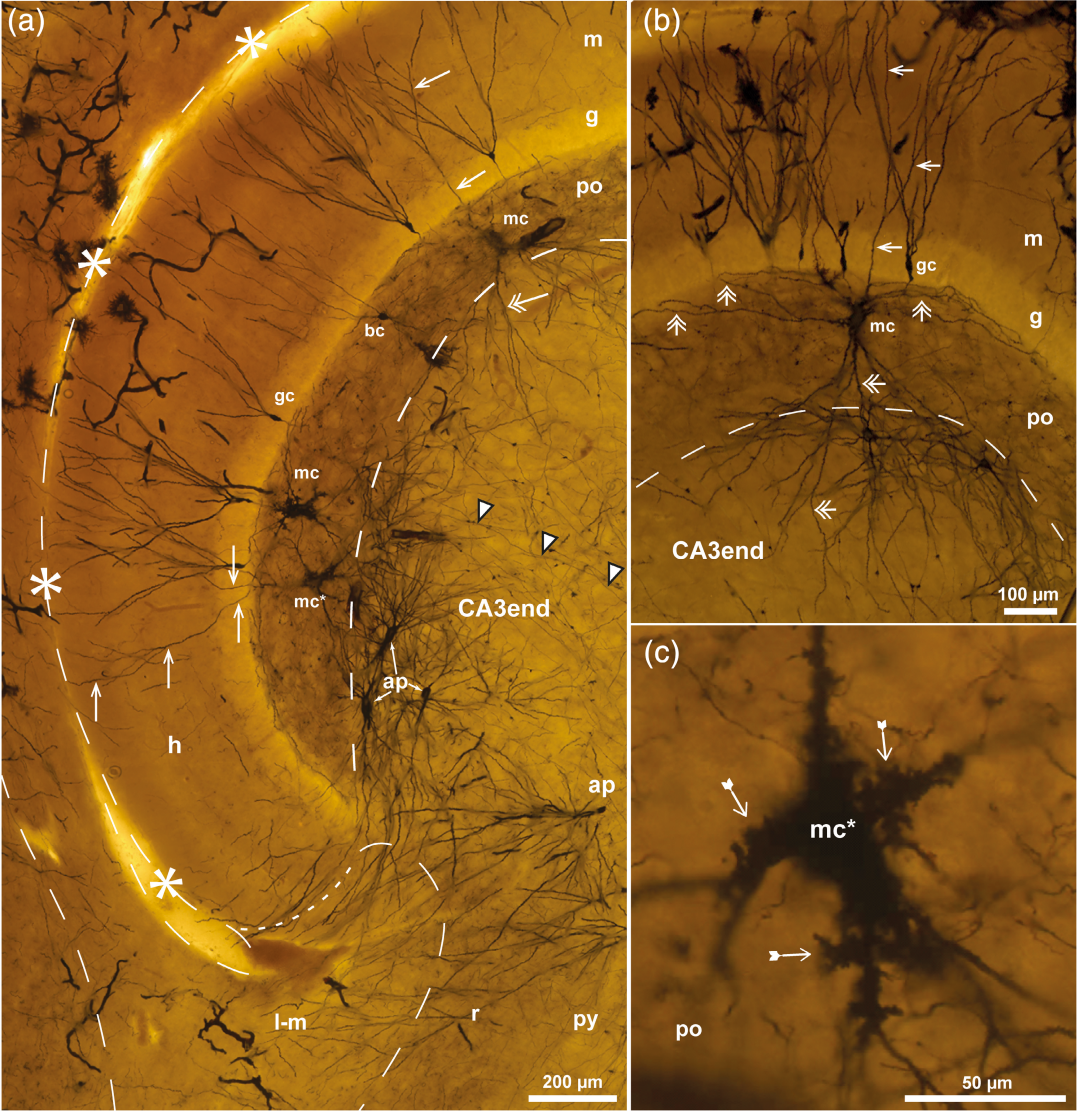
Mossy cells in sheep. Photomicrographs of a Golgi‐stained section of the DG cut perpendicular to the long septotemporal axis of the hippocampus at a temporal level. (a) Represents the boxed area in Figure 3a with three impregnated mossy cells (mc) and one basket cell (bc) in the polymorphic layer (po) and four apical pyramidal cells (ap) in the subjacent CA3 end with dendrites entering the lacunar‐molecular layer (l‐m). (b) exhibits a single mc with the soma in the po, a gm‐dendrite (arrows) ascending to the granular (g) and molecular layers (m) and distal dendrites (double arrows) coursing in the subgranular zone or entering the subjacent CA3 end where they intertwine with the dendrites of two small CA3‐interneurons respecting the border with the po. (c) Higher magnification of the mossy cell marked mc* in (a). Excrescences are marked by tailed arrows, mossy fibers by white arrowheads, the hippocampal fissure by asterisks and dashes, and a loop in the end, mossy fibers by white arrowheads, the po/CA3 border and the l‐m by dashes apart from the l‐m/m border which is dotted. gc, granule cell; h, hidden blade; py, pyramidal layer; r, radiate layer.

The mossy fiber collateral plexus in our Timm‐stained sections of sheep appeared almost homogeneously black, occupying the polymorphic layer, except for the Timm‐negative subgranular zone, which appeared very slim, particularly in the exposed blade where it was barely visible (Figure 2). The plexus exhibited distinct, but somewhat grainy borders with occasional minor excavations next to the CA3 (Figure 2d,e). Within the CA3 end, the neuropil appeared studded with black, irregularly shaped mossy fiber giant terminals (Figure 2d,e, black arrowheads), which at all hidden‐exposed levels were less numerous in the cell poor zone than in the cell rich zone. In the latter site, the terminals tended to be oriented in rows apparently representing the otherwise unstained mossy fibers converging toward the dense mossy fiber bundle on the radiate side of the pyramidal layer (Figure 2b; see also Holm & Geneser, 1991b, their fig. 6), consistent with the bundling of unmyelinated thin fibers observed electron microscopically in rat both in the deep polymorphic layer (Laatsch & Cowan, 1966) and in the mossy fiber bundle (Blackstad & Kjaerheim, 1961). The rows of fibers with giant terminals were apparently orientated in the perpendicular plane, as previously shown for the fibers of the densely packed mossy fiber bundle in rat (Acsády et al., 1998; Blackstad et al., 1970). At the end of the mossy fiber bundle and the corresponding stratum lucidum, the CA3–CA2 border is marked by a bend of the pyramidal layer (Figure 2a) also shown in the rat (Ishizuka et al., 1990, his figs. 1–4). In contrast to the abrupt turn of the mossy fiber bundle in the temporal direction at the CA3–CA2 border in rat (Cappaert et al., 2015; Gaarskjaer, 1978) and monkey (Kondo et al., 2008), some mossy fibers (in both of our sheep) fan out at the border while others continue as a narrow bunch into CA2 and part of CA1 (Figure 2b). Similar observations have previously been reported in hedgehog (Gaarskjaer et al., 1982).

In Golgi‐stained sections, the brighter granular layer contrasted with the darker polymorphic layer (Figure 3). The few impregnated granule cells varied in soma size and dendritic thickness (Figure 3b), in agreement with previous reports in mink (Blackstad et al., 2016) and monkey (Seress & Frotscher, 1990), as well as with personal observations (K.K. Osen) in Golgi‐stained sections from rat, guinea pig, and cat. The mossy fiber collateral plexus, which featured a vaguely fibrillar texture, seemed to have a more distinct border with the CA3 than with the subgranular zone (Figure 3b). Mossy fibers could be traced from the base of the impregnated granule cells, but were lost to view in the collateral plexus, to occur again scattered at its deep border with the CA3 end within which they continued without further branching. In their initial course within CA3, the fibers had only few giant terminals (Figure 3b) in accordance with the scarcity of Timm‐positive structures in the cell poor zone (Figure 2). The uniform appearance of this zone throughout its entire hidden‐exposed span in Timm‐stained sections, indicates an evenly spaced transition of mossy fibers from the entire collateral plexus.

#### 3.2.3 Mossy cells of the sheep DG in Golgi staining

Mossy cells represent the major glutamatergic (excitatory) type of neuron in the polymorphic layer (Amaral, 1978; Scharfman, 2018; Scharfman & Myers, 2012). The few impregnated mossy cells in the Golgi‐stained sections of sheep, featured short, thick primary dendrites studded with large excrescences (Figure 4c), and a brush‐like type of arborization with long straight, unbranched distal dendrites (Figure 4a,b). The excrescences were located closer to the soma than in mink (Blackstad et al., 2016, their fig. 2c) and monkey (Buckmaster & Amaral, 2001, their figs. 3a and 4c), possibly also encroaching upon the soma, but considerably less so than in hamster (Murakawa & Kosaka, 2001). In the Golgi‐stained fetal sheep material of Godina and Barasa (1964), no mention is made of mossy cells with excrescences, but also in human the excrescences do not develop until after birth (Seress, 2007; Seress & Mrzljak, 1992). While the somata and excrescence‐covered proximal dendrites of mossy cells in sheep appeared confined to the territory of the mossy fiber collateral plexus, in agreement with the synapses of large collateral terminals with the mossy cell excrescences in rat (Acsády et al., 1998; Scharfman & Myers, 2012), many distal mossy cell dendrites clearly extended outside the plexus, as also shown previously in mink (Blackstad et al., 2016). On approaching the granular layer, the mossy cell dendrites tended to bend and continue beneath the granular layer (Figure 4b), as also shown in guinea pig (Scharfman & Schwartzkroin, 1988) and mink (Blackstad et al., 2016), running partly within the subgranular zone. At least one dendrite per cell ascended into the granular and molecular layer as a typical gm‐dendrite (Figure 4a,b), in agreement with observations from several other species (guinea pig: Blackstad, 1985; rat: Scharfman, 1991; Buckmaster et al., 1993; Buckmaster, 2012; monkey: Buckmaster & Amaral, 2001; mouse: Kowalski et al., 2010; mink: Blackstad et al., 2016; human: Seress & Mrzljak, 1992). The exact number of gm‐dendrites per cell in sheep could not be determined, but it is clearly less than in human (where the mean number is 5 per cell; Buckmaster & Amaral, 2001) and in mink (mean = 2.8 per cell; Blackstad et al., 2016), although with a large span in both species. In rat, only a few mossy cells exhibit a gm‐dendrite (Buckmaster, 2012). At the deep border of the polymorphic layer, many distal mossy cell dendrites continued into the subjacent CA3 end (Figure 4a,b), as also observed in monkey (Buckmaster & Amaral, 2001, their figs. 4 and 5) and mink (Blackstad et al., 2016, their figs. 1e and 9), but considerably deeper in sheep than in mink.

#### 3.2.4 Pyramidal cells of the sheep CA3 end in Golgi‐staining

**FIGURE 5:**
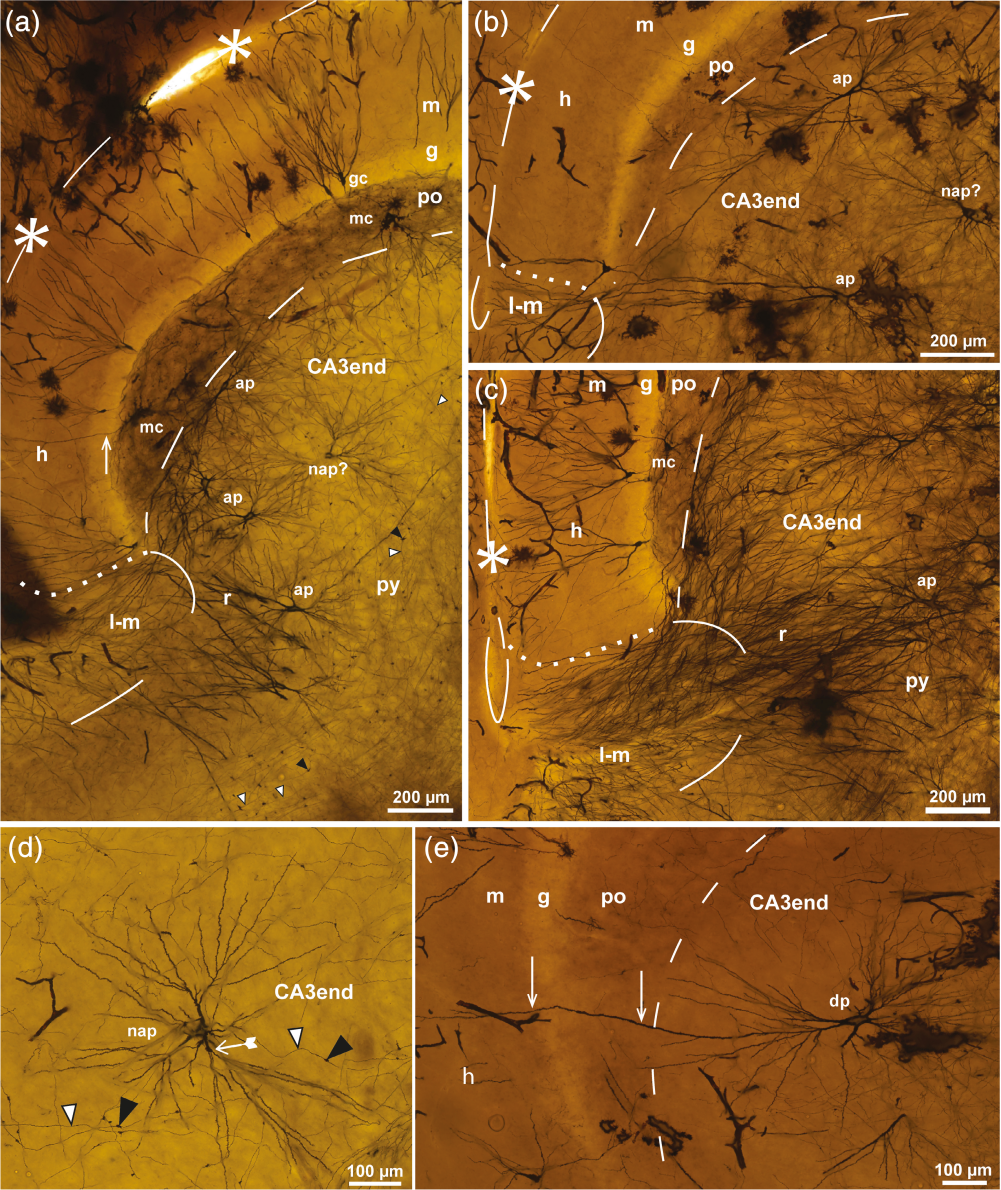
Pyramidal cells in sheep. Photomicrographs of Golgi‐stained sections of the DG cut perpendicular to the long septotemporal axis of the hippocampus at temporal (a, c, e), middle (b), and septal (d) levels. The section in (a) is adjacent to that of Figure 4a, and features the hidden blade (h) of the DG with part of the CA3 end. A gm‐dendrite of a mossy cell is marked by a white arrow. In (a–c) (the latter representing a heavily impregnated section form the surface of a tissue block) apical dendrites of apical pyramidal cells (ap) reach the lacunar‐molecular layer (l‐m). (d) Single nonapical pyramidal cell (nap) from the middle of the CA3 end with proximal dendrites covered by small excrescences (tailed arrow). A possible nap in a is marked “nap?”. (e) Dentate pyramidal cell (dp) extends a gm‐dendrite (white arrows) through the polymorphic layer (po), granular layer (g), and molecular layer (m) of the h. The hippocampal fissure is marked by asterisks and dashes and a loop in the end, mossy fibers by white arrowheads, giant terminals by black arrowheads, the po/CA3 border and the l‐m by dashes apart from the dotted l‐m/m border. gc, granule cell; mc, mossy cell; py, pyramidal layer; r, radiate layer.

The majority of Golgi‐impregnated cells within the CA3 end were found to meet the criteria of pyramidal cells with a brush‐like type of arborization with long straight distal dendrites slightly thicker than in mossy cells and with postsynaptic excrescences that were smaller and located farther from the soma, usually around the first and second dendritic branching point (Figure 5d), as also shown by Ishizuka et al. (Ishizuka et al., 1995, their fig. 1) in the DG‐near end of the CA3 pyramidal layer in rat. In accordance with the intracellular labeling study by Buckmaster and Amaral (2001) in monkey, we found three subtypes of pyramidal cells in sheep: apical, nonapical and dentate.

*Apical pyramidal cells* (called classical by Buckmaster & Amaral, 2001) with a bipolar soma and apical dendrites reaching the lacunar‐molecular layer like CA3 pyramidal cells in general (Blackstad, 1985; Ishizuka et al., 1995, their figs. 1 and 15), occurred near the hidden blade (Figure 5a–c). In Figure 5c, from the surface of a sheep tissue block with heavy impregnation, numerous apical dendrites were seen to converge toward the U‐turn of the lacunar‐molecular layer while clearly avoiding the polymorphic layer, reminiscent of the classical Golgi‐drawing of Lorente de Nó (1934, his fig. 24) from the monkey hippocampus.

*Nonapical pyramidal cells*, appearing more or less multipolar without dendrites to the lacunar‐molecular layer (Figure 5d), seemed to predominate in the exposed part of the CA3 end, but might also be scattered between the apical pyramidal cells in the hidden part (Figure 5a). Apical dendrites of these cells might have gone undetected, but it is reason to doubt whether dendrites could reach the long distance from the exposed extremity of the CA3 end to the lacunar‐molecular layer.

*Dentate pyramidal cells*, characterized by one or more dendrites extending into the polymorphic, granular and molecular layers (Figure 5e), like gm‐dendrites of mossy cells, were rarely found. They typically occurred in the hidden blade, but were also observed in the exposed crest. Near the end of the hidden blade, where the borders of the granular and molecular layers with the lacunar‐molecular layer sometimes appeared indistinct, it was less obvious whether a pyramidal cell should be classified as apical or dentate.

Since both the molecular and the lacunar‐molecular layers are supplied by the perforant path (Blackstad, 1956, his fig. 13), the nonapical pyramidal cells may be the only of the three subtypes that lacks a direct access to perforant path fibers, while only the dentate pyramidal cells traverse the border with the polymorphic layer to reach these afferents.

### 3.3 Cyto‐ and fibro‐architecture of the polymorphic layer and CA3 end in domestic pig

**FIGURE 6:**
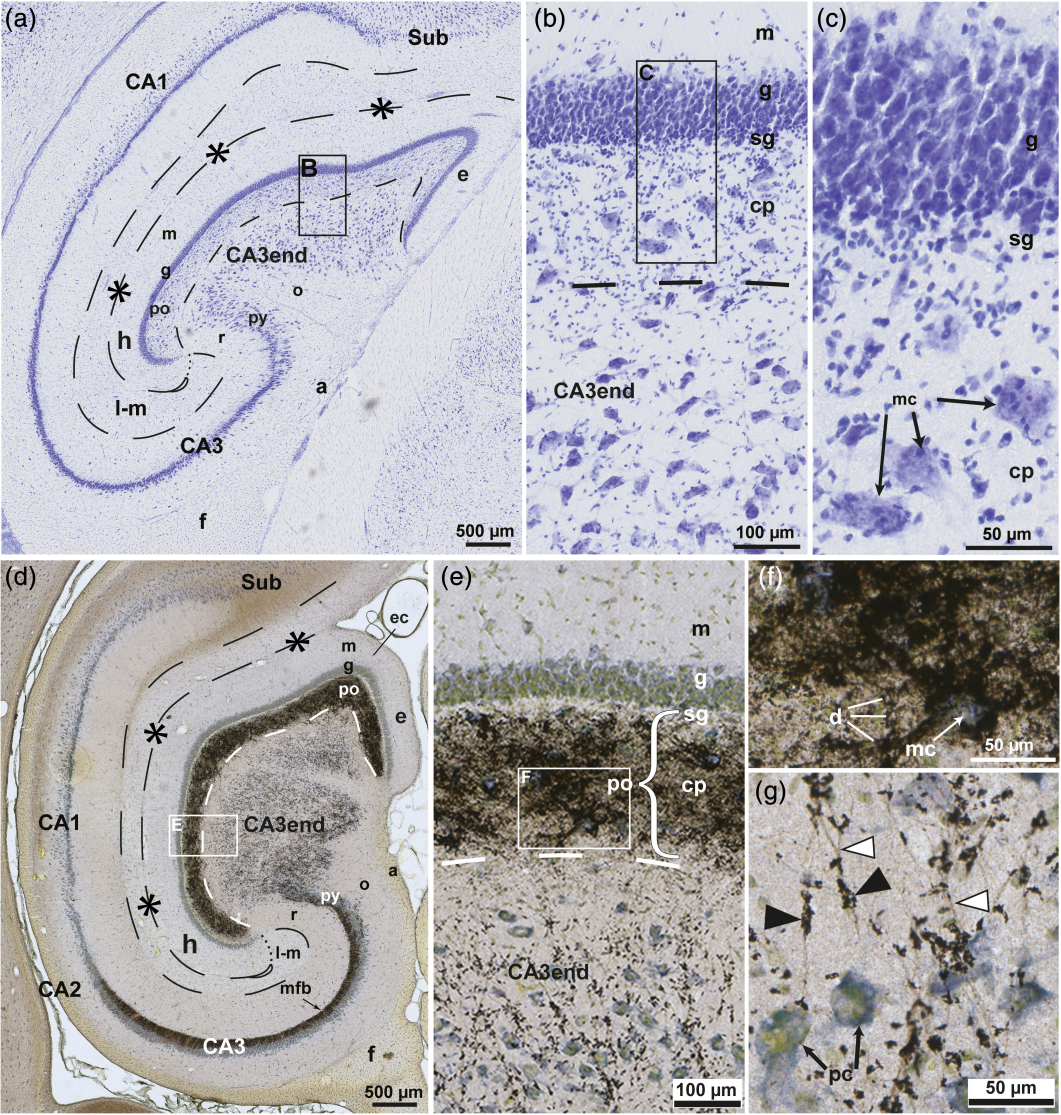
The pig dentate gyrus in thionin‐ and Timm–thionin‐staining. Photomicrographs of thionin‐(a–c) and Timm–thionin‐(d–g) stained sections probably cut in the horizontal plane at nonspecified levels. Boxed area in (a), (b), (d), and (e) are shown at higher magnification in (b), (c), (e), and (f), respectively. A Timm‐negative subgranular zone (sg) of the polymorphic layer (po) is present in all blades. The hippocampal fissure is marked by asterisks and dashes, and a loop in the end, the po/CA3 border and the lacunar‐molecular layer (l‐m) by dashes apart from the dotted l‐m/molecular layer (m) border, mossy fibers by white arrowheads, and giant terminals by black arrowheads. a, alveus; CA1–CA3, cornu ammonis area 1–3; cp, collateral plexus; e, exposed blade; ec, exposed crest; f, fimbria; g, granular layer; h, hidden blade; mf, mossy fiber bundle; mc, mossy cell; o, oriens layer; pc, pyramidal cell; py, pyramidal layer; r, radiate layer.

**FIGURE 7:**
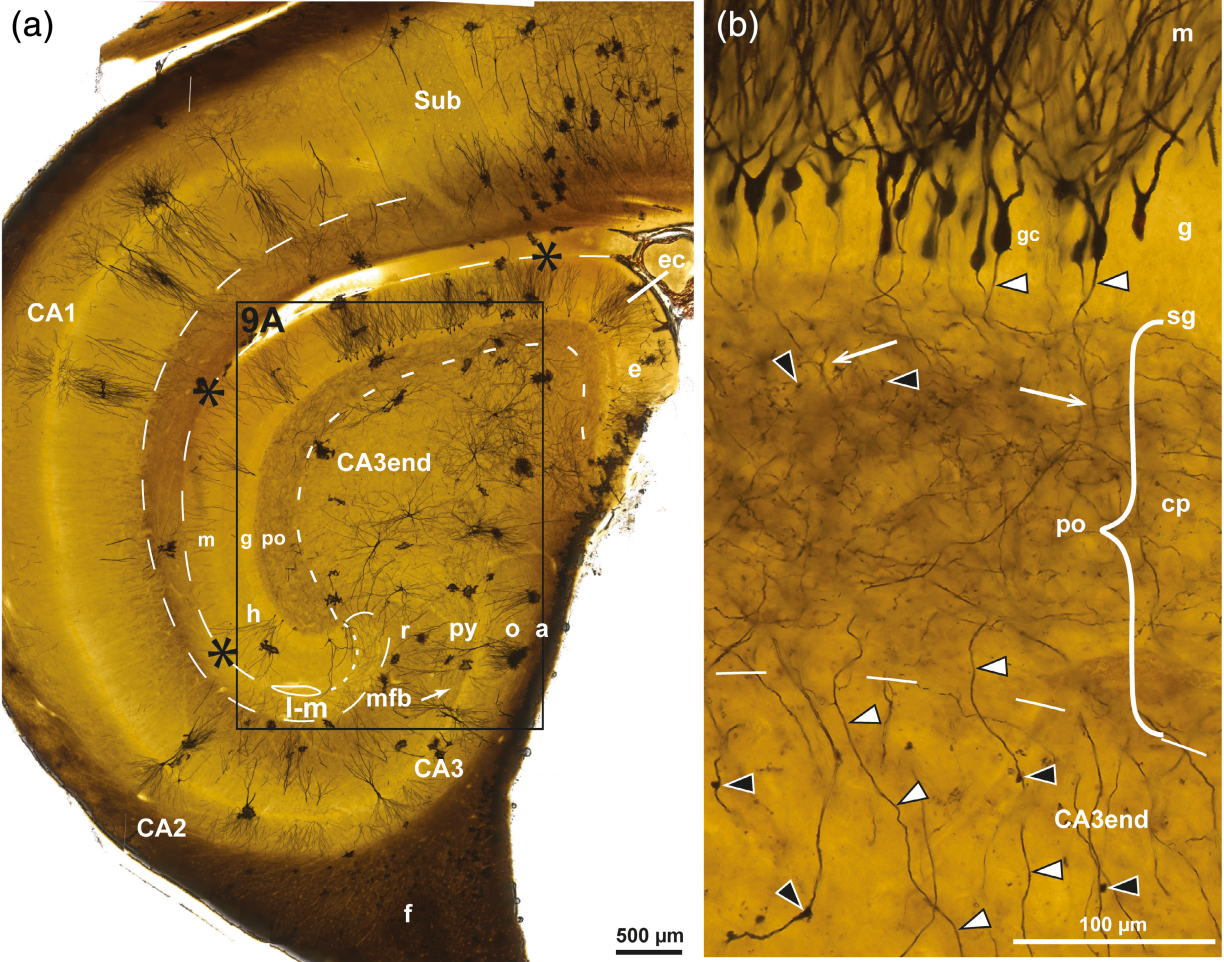
Mossy fiber collateral plexus in Golgi‐sections of pig. Photomicrographs of Golgi‐stained sections of the DG cut perpendicular to the long septotemporal axis of the hippocampus at midlevel. Boxed area in (a) is reproduced in Figure 9. (b) Detail with impregnated granule cells (gc), mossy fibers (white arrowheads) that give off collaterals (branching points indicated by white arrows) within the collateral plexus (cp), but not within the CA3 end. Giant terminals (black arrowheads) are smaller in the cp than in the CA3 end. The hippocampal fissure is marked by asterisks and dashes, and a loop in the end, the po/CA3 border and the lacunar‐molecular layer (l‐m) by dashes apart from the dotted lacunar‐molecular/molecular (l‐m/m) border. a, alveus; CA1– CA3, cornu ammonis area 1–3; e, exposed blade; ec, exposed crest; f, fimbria; g, granular layer; h, hidden blade; o, oriens layer; po, polymorphic layer; py, pyramidal layer; r, radiate layer.

**FIGURE 8:**
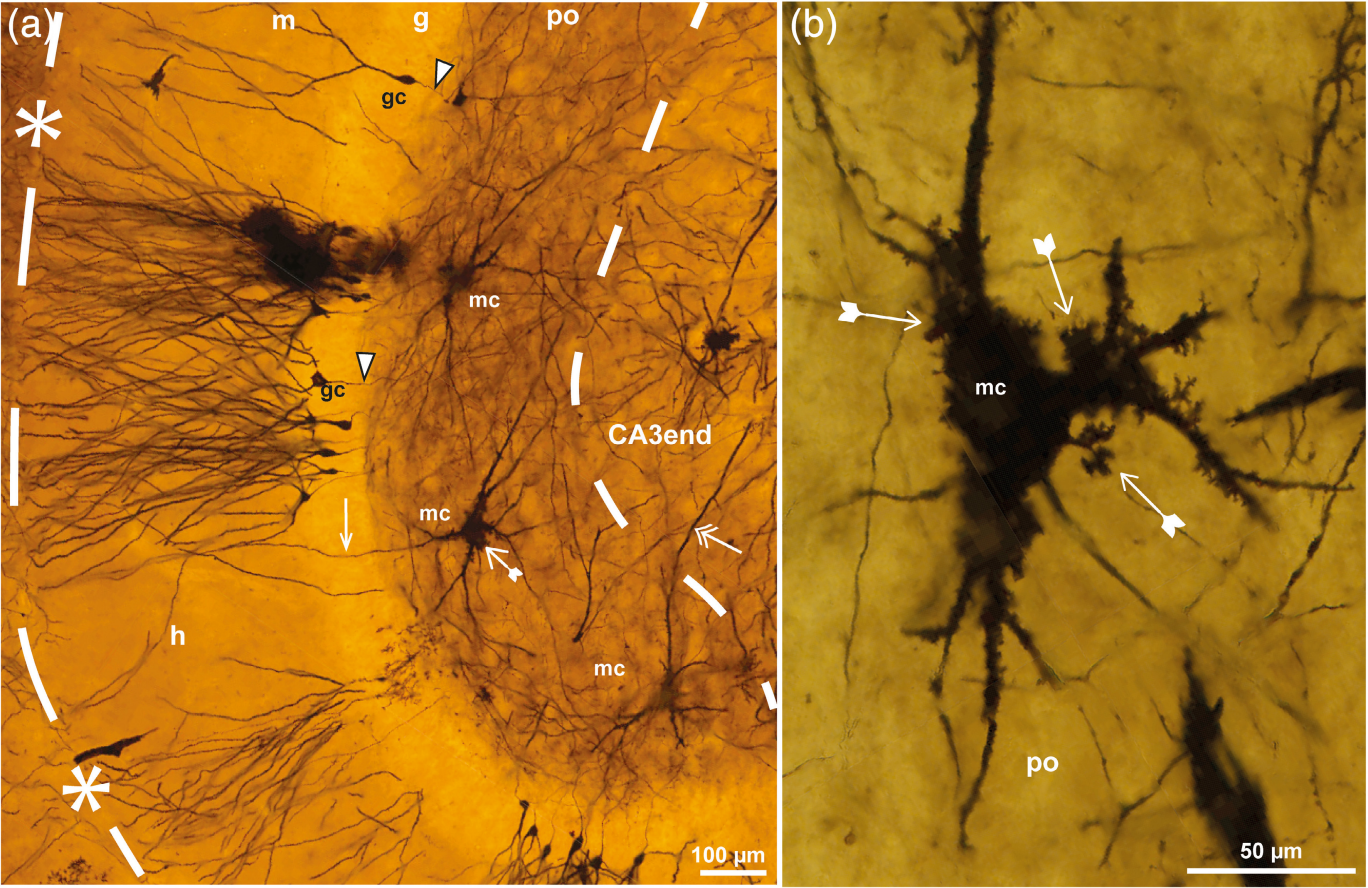
Mossy cells in pig. Photomicrographs of Golgi‐stained sections cut perpendicular to the long septotemporal axis of the hippocampus at midlevel. (a) Features three mossy cells (mc) in the polymorphic layer (po), one of which with a gm‐dendrite (arrow) ascending to the granular (g) and molecular (m) layers, another with a distal dendrite (double arrow) descending to the CA3 end. (b) A single mc at higher magnification with large excrescences (tailed arrows) on primary and secondary dendrites and soma. The hippocampal fissure is marked by asterisks and dashes, the border of the lacunar‐molecular layer (l‐m) and the po/CA3 border by dashes. gc, granule cell; h, hidden blade.

**FIGURE 9:**
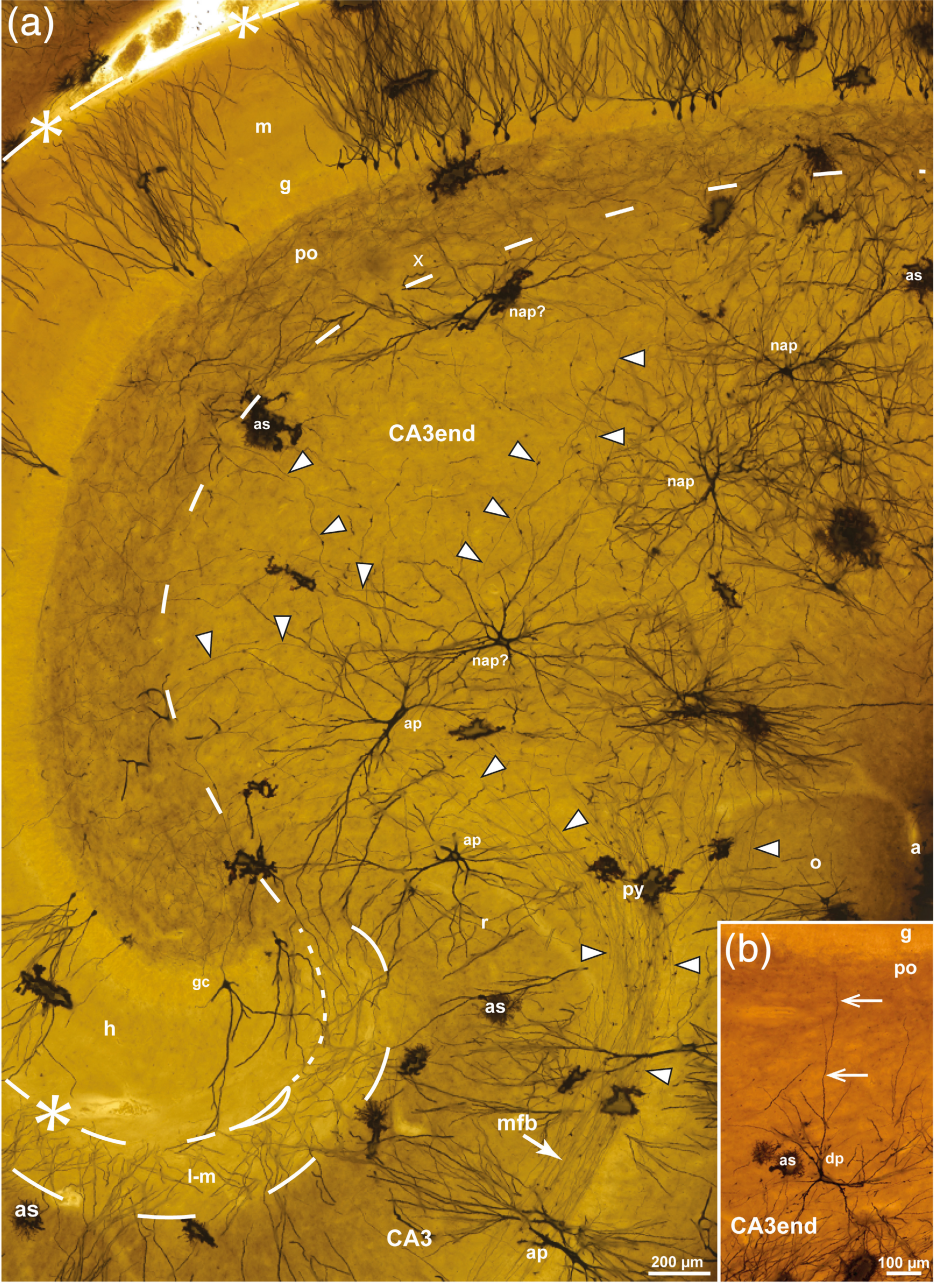
Pyramidal cells in pig. Photomicrographs of Golgi‐stained sections cut perpendicular to the long septotemporal axis of the hippocampus at middle (a) and septal (b) level. A: Higher magnification image of the boxed area in Figure 7a with apical (ap) and nonapical (nap) pyramidal cells in the CA3 end. The hippocampal fissure is marked by asterisks and dashes and a loop in the end, the po/CA3 border and the lacunar‐molecular layer (l‐m) by dashes apart from the dotted border with the molecular layer (m), mossy fibers by white arrowheads. (b) Dentate pyramidal cell (dp) with a single dendrite (arrow) penetrating the polymorphic layer (po) of the middle blade. a, alveus; as, astrocyte; CA3, cornu ammonis area 3; g, granular layer; gc, granule cell; h, hidden blade; mf, mossy fiber bundle; o, oriens layer; py, pyramidal layer; r, radiate layer; x, excavation.

Our observations were made in Golgi‐, thionin‐, and Timm–thionin‐stained pig brain sections. The Golgi‐stained sections (Figures 7, 8, 9) were oriented in the perpendicular plane, while the thionin‐ and Timm–thionin‐sections (Figure 6) were cut in a conventional horizontal plane, as in the pig studies of Holm and Geneser (1989, 1991a, 1991b), resulting in a slightly different shape and intrinsic structure of the DG and the CA3 end (compare Figures 2a and 6a,d). The main pattern of layering, nevertheless, appeared principally similar.

#### 3.3.1 Cytoarchitecture of the pig DG and CA3 end in thionin‐staining

In the thionin‐stained sections, the mossy cell somata of the polymorphic layer appeared located deep to the subgranular zone (Figure 6a–c), as in sheep. Due to the better tissue preservation, the cell rich neuropil of the polymorphic layer appeared more striking, particularly in the subgranular zone where numerous minute cells adjacent to the basalmost granular cells (Figure 6b,c) might have included both glial cells with processes to the granular layer as shown in monkey (Eckenhoff & Rakic, 1984, their fig. 10), and granule cell precursors as shown in rabbit (Guéneau et al., 1982). In the CA3 end, the transition between the cell rich and the cell poor zone appeared less distinct than in sheep. Next to the hidden blade, there was a slight tendency for pyramidal cell somata to be elongated and oriented in parallel across the continuation of the curved long axis of the CA3 pyramidal layer, as also seen in sheep, although less clearly, a difference that might be ascribed to the different planes of sectioning (compare Figures 2a and 6a).

#### 3.3.2 The mossy fiber system of pig in Timm‐ and Golgi‐staining

As in sheep, the band‐shaped polymorphic layer in pig exhibited a Timm‐positive collateral plexus covering the mossy cell somata, as well as a thin Timm‐negative subgranular zone (Figure 6d,e). In accordance with the better tissue preservation, the plexus at higher magnification of the Timm‐stained sections, featured a grainy texture compatible with stained tiny terminals, as well as indistinct black silhouettes of large multipolar cells with an unstained center, most likely representing somata and proximal dendrites of mossy cells surrounded by Timm‐positive collateral terminals (Figure 6e,f). Deep to the exposed crest, a few such cells also appeared immediately deep to the polymorphic layer (Figure 6d). Small bright spots surrounded by black material in the plexus likely represent crosscut dendrites surrounded by afferents (Figure 6f, marked d), while the serrated appearance of the borders of the plexus as a whole reflected cellular processes covered in Timm‐positive terminals.

As in sheep, the cell poor zone of the CA3 end exhibited fewer Timm‐positive mossy fiber giant terminals than the cell rich zone (Figure 6d,e). Within the cell rich zone, the giant terminals, which here appeared connected by tiny, faintly stained fibers, were clearly located *between* the cell bodies (Figure 6e,g), rather than enveloping the somata as seen for mossy cells within the collateral plexus. The observation is consistent with the location of the pyramidal cell postsynaptic excrescences farther from the cell body (Ishizuka et al., 1995) than in the mossy cells, but not on the distal dendrites which apparently constitute the major part of the cell poor zone.

In the available Timm‐section of the pig hippocampus, the CA3 differs from that of sheep both in shape and texture (compare Figures 2a and 6d). The differences are most probably due the different planes of sectioning. Whereas in the perpendicular sections in sheep, the Timm‐positive terminals appeared in rows converging toward the CA3 mossy fiber bundle, in the horizontal section in pig, the terminals were collected in randomly arranged, tiny groups that were larger closer to the main CA3, where only the most hidden part of the CA3 end appeared continuous with the pyramidal layer and mossy fiber bundle. In the exposed part, instead, the CA end formed a few wide protrusions toward the oriens layer, supposedly reaching the pyramidal layer at other levels. The random distribution of the groups of terminals in the horizontal plane is compatible with the orientation of the mossy fibers in the perpendicular plane.

In Golgi‐sections from pig, the collateral plexus of the polymorphic layer featured an irregular network of tiny branching collaterals with scattered small “giant” terminals (Figure 7b) and *én passant* varicosities, which were visible only at high magnification. Because the impregnated granule cells tended to occur in clusters, it was not possible to identify the collateral tree of single mossy fibers. As in sheep, and in agreement with our Timm‐sections and the drawings of Ramon y Cajal (1911, his figs. 478 and 482) from guinea pig and rabbit, the main mossy fibers with occasional giant terminals were found to leave the deep border of the plexus in a scattered formation, to course within the CA3 end (Figures 7b and 9).

#### 3.3.3 Mossy cells of the pig DG in Golgi‐staining

In pig, as in sheep, thionin‐stained mossy cell bodies appeared slightly larger than those of pyramidal cells (Figure 6b). In Golgi‐sections, the mossy cell excrescences were as prominent as in sheep, being located on the soma and secondary dendrites as well as on primary dendrites (Figure 8b). As in sheep, the mossy cells had at least one gm‐dendrite each ascending to the granular and molecular layers, while distal dendrites were seen to enter the CA3 end (Figure 8a).

#### 3.3.4 Pyramidal cells of the pig CA3 end in Golgi‐staining

In Golgi‐sections from pig, as in sheep, apical pyramidal cells with dendrites to the lacunar‐molecular layer were found next to the hidden blade, while nonapical pyramidal cells appeared to dominate near the exposed blade (Figure 9a). In our relatively restricted Golgi‐material, we only found a single cell that may represent an incomplete dentate pyramidal cell with a dendrite penetrating into, but not beyond the polymorphic layer (Figure 9b). Overall, the dendrites of the pyramidal cells, with the exception of the dentate subtype, seemed to respect the border with the polymorphic layer/collateral plexus. Close to the border with the polymorphic layer, the pyramidal cells seemed to adapt the shape of their dendritic tree to the border, occasionally forming an excavation into the DG polymorphic layer (marked “x” in Figure 9a). However, as also demonstrated in the Timm‐stained sections, a borderline composed of cellular elements can hardly be strictly linear.

## 4 DISCUSSION

**FIGURE 10:**
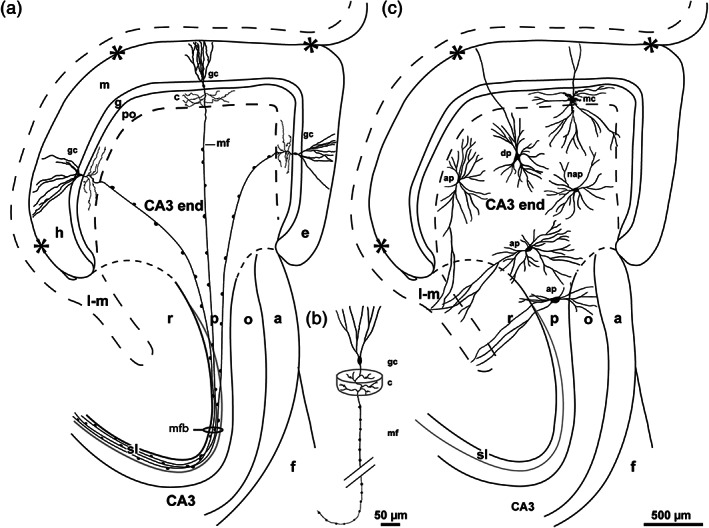
Summary diagram of the dentate gyrus and the subjacent CA3 end in sheep and pig in sections cut perpendicular to the long, septotemporal axis of the hippocampus, with focus on: (a) The mossy fiber system with its origin in the granule cells (gc), the collaterals in the polymorphic layer (po), and the main mossy fibers featuring regularly spaced giant terminals and converging through the CA3 end toward the mossy fiber bundle (mfb) in the main CA3. (b) A single gc with mossy fiber (mf) and collaterals (c) seen in a slightly different view in order to emphasize the 3‐D shape and orientation of the collateral tree. (c) The position of mossy cells (mc) with soma in the po and of the three pyramidal cell subtypes, i.e., the apical pyramidal cells (ap), dentate pyramidal cells (dp), and nonapical pyramidal cells (nap) in the CA3 end. The hippocampal fisssure is marked by asterisks and a loop in the end, the po/CA3 border, the lacunar‐molecular layer (l‐m) and the stratum lucidum (sl) by dashes. The end of the visibly laminated stratum radiatum, stratum oriens, and alveus is marked by stippled lines. Scalebar in (c) applies also to (a). a, alveus; e, exposed blade; f, fimbria; g, granular layer; h, hidden blade; l‐m, lacunar‐molecular layer; m, molecular layer; o, oriens layer; p, pyramidal layer; r, radiate layer.

We have studied the DG and CA3 end of sheep and domestic pig, with focus on the DG/CA3 border defined by the mossy fiber system. We compared Timm–thionin‐ and Golgi‐stained sections, which in sheep were from adjacent blocks of the same hippocampus cut perpendicular to its long curved axis, approximately parallel to the main mossy fibers (=granule cell axons). The perpendicular (or “lamellar”) plane of sectioning highlighted the relatively simple shape and pattern of the DG and CA3 end, which because of the curved, twisted long axis of the hippocampus may appear rather confusing in conventional planes of sectioning. The main findings were largely similar in the two species and are summarized in a common diagram (Figure 10), that features the long and slim polymorphic layer, with mossy fiber collaterals and mossy cells, capping the expanded CA3 end, containing scattered mossy fibers and pyramidal cells. A single granule cell with mossy fiber and collaterals is shown in a slightly different view to visualize the “nonlamellar” orientation of the collateral tree.

Notably, our Figure 10 resembles the otherwise much more sophisticated and 110 years older diagram of the Ammon's horn by Ramon y Cajal (1911, 1955, his fig. 479) in young rabbits, both with respect to the mossy fiber collaterals in the polymorphic layer and the convergence of the scattered main mossy fibers through the CA3 end. Ramon y Cajal's diagram, however, just missed the expansion of the CA3 end, and the three other topics that were central in our study, namely a DG/CA3 borderline, mossy cells in the polymorphic layer, and pyramidal cells of various subtypes in the CA3 end.

### 4.1 The comparative architecture of the polymorphic layer with its mossy fiber collateral plexus

With its long, band‐shaped polymorphic layer capping the expanded CA3 end, the DG in sheep and domestic pig (Figure 10) resembles the DG in monkey (Buckmaster & Amaral, 2001; Kondo et al., 2008) and human (Cassell & Brown, 1984). It is distinctly different from the polymorphic layer in rat, which exhibits a triangular cross‐section filling the larger part of the so‐called hilus deep to the molecular and granular layers, abutting on the end of the pyramidal layer of the CA3 (Boccara et al., 2015; Kjonigsen et al., 2015), and the adjacent radiate and oriens layers. Judged by our material and consistent with observations in other mammals (guinea pig: Geneser‐Jensen et al., 1974; rabbit: Geneser, 1987a, Geneser, 1987b), the polymorphic layer in sheep and pig consists of a minor Timm‐negative subgranular zone and a major Timm‐positive zone corresponding to the mossy fiber collateral plexus which according to our definition constitutes the border of the polymorphic layer with the subjacent CA3 end. The two zones correspond to the outer plexiform layer and the outer hilar cell layer, respectively, of the so‐called “area dentata” defined by Holm and Geneser (1991b) in pig.

*The mossy fiber collaterals* were observed already by Camillo Golgi in 1886. The mossy fibers and their collateral plexus were illustrated in more detail by Ramon y Cajal (1911, his figs. 478, 479, and 482) in Golgi‐stained sections of rabbit and guinea pig. Although Ramon y Cajal labeled the collateral plexus as “*l'assise des cellules polymorphes*”, it is not clear if he regarded the plexus to define the border of the polymorphic layer with the CA3, as concluded by us. He did not, however, have at hand the Timm‐method, which was developed half a century later (Timm, 1958), enabling identification of the collaterals by their zinc‐containing boutons, and inspiring T.W. Blackstad (1963) to name the plexus “*the Z‐zone*”. Despite the dense Timm‐staining indicating a packing density of terminals in the plexus on a par with the mossy fiber bundle, the collateral plexus has received remarkably little attention in recent literature. Noteworthy exceptions are the brilliant studies by Claiborne et al. (1986) and Acsády et al. (1998) based on intracellular labeling of granule cells in rat. According to Claiborne et al. (1986), each mossy fiber in rat gives rise to 5–12 extremely thin branching and winding collaterals about 100–350 μm long extending in all directions. The corresponding estimates of Acsády et al. (1998) are 5–8 and 400–700 μm, respectively. Consistent with our observations in Golgi‐sections from sheep and pig, both studies found the collaterals to be endowed with a few larger terminals, somewhat smaller than the giant terminals of the main fibers, as well as numerous smaller *en passant* presynaptic varicosities. Electron microscopically, the larger terminals have been found to be presynaptic to the mossy cell excrescences, while the *en passant* terminals synapse mainly on GABA‐ergic interneurons (Acsády et al., 1998; Laatsch & Cowan, 1966), including basket cells. The numbers per granule cell are according to Claiborne et al. (1986) about seven “giant” terminals (viz., about one per collateral) and 160 small varicosities; according to Acsády et al. (1998) 7–12 and 147, respectively, with some intermediate forms. In view of the high number of *en passant* terminals, the collaterals may be Timm‐positive throughout most of their length, consistent with the density of the plexus in our Timm‐stained sections.

Because in our Golgi‐sections, the impregnated granule cells tended to occur in groups, individual collateral trees could not be identified. Exact counts and measurements of the mossy fiber collaterals, therefore, were not possible. The number of four collaterals per mossy fiber, together forming a collateral tree with diameter about 400 μm, as indicated in Figure 10a,b, are only rough estimates, based on the above mentioned data in rat, adjusted by observations in Golgi‐stained sections of sheep and pig, at the periphery of areas with many impregnated granule cells. Interestingly, the estimated size and shape of the collaterals are compatible with the hippocampal scheme by Ramon y Cajal (1911, his fig. 479). The data are also, with some reservations for size, compatible with the observations of Kondo et al. (2008), who following injections of an anterograde tracer involving the granular layer in monkey, reported labeling of the mossy fiber collaterals “with a very limited spatial distribution”. Judged by their figs. 6 and 8, the plexus was strongly labeled within about 700 μm measured from the center of the injections both in the transverse and the longitudinal directions.

The intention with our Figure 10a, was to give an impression of the size of a collateral tree relative to the hidden‐exposed length of the polymorphic layer, which in sheep and pig, thus, may give space for about eight segregated collateral trees side by side, in sharp contrast to the situation in rat where a single collateral tree covers almost the entire cross‐section of the polymorphic layer at septal levels (Claiborne et al., 1986, their fig. 7). In the perpendicular plane, thus, the degree of overlap between collateral trees originating in the hidden and exposed parts of the granular layer, must be considerably less in sheep, pig, and monkey, than in rat.

*The mossy cells* in sheep and pig are largely in agreement with observations in other species (rat: Amaral, 1978; Scharfman & Myers, 2012; monkey: Buckmaster & Amaral, 2001; mink: Blackstad et al., 2016), located with their soma and excrescence‐covered proximal dendrites within the collateral plexus, while distal dendrites extend to areas outside the plexus including the granular and molecular layers (gm‐dendrites), the subgranular zone, and the CA3 end (Figure 10b). As indicated in Figure 10b, the dendritic tree of each mossy cell in sheep and pig covers about 1/6 of the hidden‐to‐exposed length of the polymorphic layer, being in the same order of magnitude as the collateral trees. In rat, each mossy cell dendritic tree, like each collateral tree, fills in about the whole cross‐section of the polymorphic layer. In view of the length of the hippocampus, the degree of overlap may be less in the septotemporal dimension, even in rat.

By their gm‐dendrites, which increase in number from rat to human, the mossy cells may get direct, monosynaptic input from the perforant path (Scharfman, 1991, 2016), in addition to the disynaptic input to the excrescences from the mossy fiber collaterals (Acsády et al., 1998; Scharfman, 1991), and the three‐synaptic back‐projection from the CA3 pyramidal cells (Buckmaster et al., 1993; Ishizuka et al., 1990; Li et al., 1994; Scharfman, 1994; Vivar et al., 2012; Wittner et al., 2007). The strong glutamatergic input from the mossy fiber collaterals to the mossy cell excrescences (Acsády et al., 1998), is potentially relevant in the context of excitotoxic death of mossy cells in epileptic rats (Blümcke et al., 1999; Blümcke et al., 2000).

The extension of distal mossy cell dendrites into the CA3 end is a feature of particular relevance to the DG/CA3 border. Such extensions may be missing in rat (Amaral, 1978), minimal in mink (Blackstad et al., 2016), and extensive in monkey (Buckmaster & Amaral, 2001) as in sheep and pig, perhaps related to the phylogenetically shrinking depth of the polymorphic layer and the “space‐filling type of arborization” (Fiala & Harris, 2012) of the mossy cells (Blackstad et al., 2016). The mossy cells, which also receive a rich supply from subcortical regions through the fimbria (Scharfman, 2016, 2018), are thought to play an integral part in the hippocampal circuitry by modulating the mossy fiber signals to the CA3 that are thought to be essential for episodic memory formation (Leutgeb et al., 2007; Myers & Scharfman, 2009; Treves et al., 2008).

*The subgranular zone* of the polymorphic layer varies much in relative depth between species, from being nonexistent in rat (Haug, 1974), to barely visible in mink (Blackstad et al., 2016), slim in human (Cassell & Brown, 1984) as in sheep and pig (present study), and about 1/3 the thickness of the polymorphic layer in guinea pig (Geneser‐Jensen et al., 1974) and rabbit (Geneser, 1987b). Interestingly, it can also be observed in monkey (Kondo et al., 2008; their fig. 2). The zone constitutes the secondary germinal matrix of adult‐born granule cells (Deshpande et al., 2013; Guéneau et al., 1982), one of the few neuronal types reproduced throughout life in mammals, which means that also the mossy fibers must in part be transitory. The histological variation in soma size and dendritic thickness of granule cells observed in sheep and pig, as well as in mink and other species (Blackstad et al., 2016), thus, could possibly relate to different functional stages of a life cycle (Brunner et al., 2014; Guéneau et al., 1982). In species with an inconspicuous subgranular zone, the germinal cells may possibly be intermingled with other cells in the polymorphic layer as during early ontogenesis (Altman & Bayer, 1990a, 1990b, 1990c).

### 4.2 Scattered fibers and cells in the CA3 end

The expanded CA3 end in sheep and pig differs from the main part of the CA3 by the absence of a dense mossy fiber bundle and a distinct pyramidal layer both of which appear gradually at the transition to the main CA3 (Figure 10a,c). The scattering of main mossy fibers in the CA3 end deep to the collateral plexus is clearly consistent with previous Golgi‐studies in guinea pig and rabbit (Ramon y Cajal, 1911, his figs. 478, 480, and 479) as well as with illustrations in previous Timm‐studies in human (Sutula et al., 1989), domestic pig (Holm & Geneser, 1989, 1991a, 1991b), and guinea pig (Blackstad, 1985; Geneser‐Jensen et al., 1974). In rat (Haug, 1974, his fig. 2e), and mink (Blackstad et al., 2016, their fig. 1f), where the CA3 is stratified to its end, the mossy fibers in Timm‐stained sections, instead, seem to enter the CA3 largely at the tip of the pyramidal layer. Nevertheles, in the intracellular HRP‐labeling experiments in rat (Claiborne et al., 1986, their fig. 14), and in Golgi‐stained sections of mink (Blackstad et al., 2016, their fig. 2a), additional main mossy fibers reach the pyramidal layer by passing through the adjacent radiate and oriens layers. The reason why these fibers do not show up in Timm‐staining, is probably that the layers are composed mainly of distal pyramidal cell dendrites without postsynaptic excrescences, a feature also reflected in the fewer Timm‐positive terminals in the cell poor zone of the CA3 end in sheep and pig compared to the cell rich zone. A scattered transition of the main mossy fibers into the CA3 end, thus, may be a rule rather than an exception.

As shown electron microscopically in rat, the mossy fibers, which are unmyelinated with a diameter <1 μm, tend to form minor bundles both within the deep portion of the polymorphic layer (Laatsch & Cowan, 1966) and the mossy fiber bundle (Blackstad & Kjaerheim, 1961). Despite the scattering of the mossy fibers in our Golgi‐sections, a certain tendency to bundling might explain the distribution pattern of Timm‐positive terminals within the CA3 end and the transitional region reminiscent of the infra‐ and supra‐pyramidal bundles in rat (Cappaert et al., 2015).

*The scattering of the pyramidal cells* in the CA3 end in sheep and pig is clearly related to the scattering of mossy fibers (Figure 10a,c). The distribution of the pyramidal cells as well as the existence of three subtypes, resemble the pattern reported in monkey (Buckmaster & Amaral, 2001). It is unknown, however, whether the hidden‐exposed gradient in the distribution of the apical and nonapical subtypes in sheep and pig applies also to monkey. The apical and dentate pyramidal cells, besides the disynaptic input through the mossy fibers, may also get monosynaptic input from the entorhinal cortex, the apical subtype to their dendrites in the lacunar‐molecular layer of the CA3, and the dentate subtype to their gm‐dendrites in the molecular layer of the DG (Ramon y Cajal, 1911; Blackstad, 1956, his fig. 13). The nonapical pyramidal cells, on the contrary, which dominate in the exposed part of the CA3 end, do not have dendrites to layers directly supplied by perforant path fibers, and thus may get only disynaptic input from the entorhinal cortex through the mossy fibers. With the exception of the dentate subtype, the pyramidal cells in the CA3 end of sheep and pig respect the border with the collateral plexus, like pyramidal cells in rat (Acsády et al., 1998; Amaral, 1978; Ishizuka et al., 1995) and mink (Blackstad et al., 2016).

It is reasonable to assume that the scattering of mossy fibers and pyramidal cells, coupled with the differentiation of the latter into subtypes, may promote a differentiated response to the perforant path signals. The conditions for a differentiated response may be brought a step further in the human brain, where in addition to the larger CA3 end, the granular and molecular layers of the DG become elongated and heavily folded (Cassell & Brown, 1984; Sutula et al., 1989) in accord with the term *fascia dentata* often used for the molecular and granular layers combined also in nonprimate species (Blackstad, 1956, 1958).

By its loose packing of the pyramidal layer and concomitant splitting of the mossy fiber bundle, the transitional region from the CA3 end to the main CA3 in sheep resembles the DG near end of the pyramidal layer in rat, referred to as CA3c (Lorente de Nó, 1934). In sheep, the CA3 end and the transitional region together constitute about 2/3 the total length of the CA3, while the remaining 1/3 exhibits a dense pyramidal layer with a compact mossy fiber bundle. In human, the CA3 which also appears relatively shorter than in rat, is made up entirely of an expanded CA3 end and a loose pyramidal layer with only traces of a dense mossy fiber bundle (Cassell & Brown, 1984; Sutula et al., 1989). In view of the various subtypes of pyramidal cells described by Amaral (Amaral, 1978, his fig. 1a) in his looser zone 1 of the rat pyramidal layer, it is tempting to regard this as a precursor of the expanded CA3 end.

### 4.3 Geometry of granule cell systems

The so‐called lamellar organization of the hippocampal mossy fibers (Andersen et al., 1969; Andersen et al., 1971; Blackstad et al., 1970), in the sense of a parallel orientation of the main granule cell axons, is a feature shared with the granule cell systems in the cerebellum (see, e.g. Voogd & Glickstein, 1998; D'Angelo, 2018) and the cochlear nuclear complex (Oertel & Young, 2004; Yaeger & Trussell, 2015). The three systems are all characterized by a large amount of minute granule cell bodies exhibiting unramified, unmyelinated axons endowed with é*n passant* synapses, and coursing in parallel perpendicular to the target neurons, which are also oriented in parallel, thus forming a strict geometrical pattern. While in all three sites, the granule cell somata are situated deep to the molecular layer, the location of the granule cell dendrites and axons with target cells differs. In the cerebellum and the cochlear nuclear complex (which both originate from the rhombic lip; see, e.g. Leto et al., 2016; Wullimann et al., 2011; Consalez et al., 2020), the short granule cell dendrites are situated in between the somata in the granule cell layer, while the granule cell axons and the dendrites of their target cells, Purkinje, and pyramidal cells, respectively, occupy the molecular layer (Blackstad et al., 1984; Mugnaini et al., 1980; Mugnaini, & Osen, 1980; Voogd & Glickstein, 1998). In the hippocampus, on the contrary, the granule cell dendrites extend into the molecular layer, while the granule cell axons and their target pyramidal cells with dendrites are located in the neighboring CA3 (Cappaert et al., 2015; van Strien et al., 2009). The difference might possibly relate to the phylogenetically late development of the DG granule cell system, with the granule cell dendrites growing toward the afferent perforant path fibers in the molecular layer while the granule cell axons invade the adjacent CA3 (Altman & Bayer, 1990a, 1990b, 1990c; Bayer, 1980; Donato et al., 2017).

The granule cell system of the DG is unique also by its plexus of granule cell axon collaterals in the polymorphic layer, where the target mossy cells and others are involved in a feedback projection to the granule cells (Acsády et al., 1998; Blackstad, 1956; Claiborne et al., 1986; Laurberg, 1979; Scharfman & Myers, 2012; Zimmer, 1971). In the DG, the mutual separation of granule cell somata, dendrites, and the majority of related interneurons is reflected in the higher packing density of the granule cell somata.

The granule cell systems all receive extensive input from other brain regions for integration and analysis (so‐called “pattern separation” or “filtering” of signals). In analogy with the hippocampal circuitry, they all project back to the sites of origin of their input signals.

## 5 CONCLUSION

Our findings support the notion that a DG/CA3 border defined by the plexus of mossy fiber collaterals are of general validity in mammals despite the interspecies variation in the topography of the polymorphic layer and the CA3 end. The borderline coincides largely with the generally accepted one, but it is more precise, and it is valid irrespective of whether the polymorphic layer has a triangular cross‐section as in rat (Haug, 1974) or a thin band capping the expanded CA3 end as in sheep and pig as well as in monkey (Buckmaster & Amaral, 2001) and human (Cassel & Brown, 1984; Sutula et al., 1989), or an intermediate shape as in mink (Blackstad et al., 2016). There is, however, still a need for further phylogenetic studies of the DG and CA3 end in an ontogenetic perspective with attention to the late development of granule cells, mossy cells and perforant path enabling input to the hippocampus proper from the entorhinal cortex via the DG, in addition to the direct input to the lacunar‐molecular layer of the CA3 and CA1 (Blackstad, 1956 his fig. 13; Ramon y Cajal, 1911, his fig. 479), and the original input to the CA3 from subcortical regions through the fimbria (emphasized by Scharfman, 2007).

## CONFLICT OF INTEREST

The authors state no conflicts of interest.

## Data Availability

The high‐resolution microscopic images utilized in the present study are shared via the EBRAINS research infrastructure (http://ebrains.eu) in the following two datasets, (1) Blackstad, J. S., Osen, K. K., & Leergaard, T. B. (2022). Microscopic images showing the fibro‐ and cytoarchitecture demarcating the border between the dentate gyrus and CA3 in sheep (*Ovis aries*) [Data set]. EBRAINS. https://doi.org/10.25493/RFM6-VQY, and (2) Blackstad, J. S., Osen, K. K., & Leergaard, T. B. (2022). Microscopic images showing the fibro‐ and cytoarchitecture demarcating the border between the dentate gyrus and CA3 in domestic pig (*Sus scrofa domesticus*) [Data set]. EBRAINS. https://doi.org/10.25493/Q4VY-8DC.
